# Changes in cardiorespiratory function and fatigue following 12 weeks of exercise training in women with systemic lupus erythematosus: a pilot study

**DOI:** 10.1136/lupus-2022-000778

**Published:** 2022-10-11

**Authors:** Sarfaraz Hasni, Li Rebekah Feng, Marquis Chapman, Sarthak Gupta, Anam Ahmad, Adam Munday, Mir Ali Mazhar, Xiaobai Li, Shajia Lu, Wanxia Li Tsai, Massimo Gadina, Michael Davis, Jun Chu, Zerai Manna, Shuichiro Nakabo, Mariana J Kaplan, Leorey Saligan, Randall Keyser, Leighton Chan, Lisa M K Chin

**Affiliations:** 1 Lupus Clinical Trials Unit, Systemic Autoimmunity Branch, National Institute of Arthritis and Musculoskeletal and Skin Diseases, Bethesda, Maryland, USA; 2 National Institute on Drug Abuse, National Institutes of Health, Bethesda, MD, USA; 3 Systemic Autoimmunity Branch, National Institute of Arthritis and Musculoskeletal and Skin Diseases, National Institutes of Health, Bethesda, Maryland, USA; 4 Rehabilitation Medicine Department, National Institutes of Health Clinical Center, Bethesda, Maryland, USA; 5 Biostatistics and Clinical Epidemiology service, National Institutes of Health Clinical Center, Bethesda, MD, USA; 6 Translational Immunology Section, National Institute of Arthritis, and Musculoskeletal, and Skin Diseases, National Institutes of Health, Bethesda, Maryland, USA; 7 National Institutes of Health, Bethesda, Maryland, USA

**Keywords:** exercise therapy, lupus erythematosus, systemic, quality of lIfe

## Abstract

**Objective:**

In patients with systemic lupus erythematosus (SLE), fatigue is a debilitating symptom with poorly understood pathophysiology. Cardiorespiratory dysfunction has been hypothesised as a contributor to SLE-fatigue. The purpose of this exploratory study was to examine changes in cardiorespiratory function, following an exercise training programme in women with SLE, together with patient reported outcomes and other pathophysiological measures that may underlie SLE-fatigue.

**Methods:**

Sixteen women with SLE and fatigue (Fatigue Severity Scale (FSS) ≥3) were enrolled in a supervised aerobic exercise training programme of vigorous intensity. The primary outcome was time to reach anaerobic threshold (AT-Time) during a cardiopulmonary exercise test (CPET). Secondary outcomes included changes in the 10-minute walk test (10MWT), FSS scores and the Patient Reported Outcomes Measurement Information System (PROMIS-57) survey. Mitochondrial function was assessed by the oxygen consumption rate (OCR)/extracellular acidification rate (ECAR) metabolic potential ratio.

**Results:**

Following 12 weeks of exercise training, AT-Time increased by 93±82 (mean±SD) s (p<0.001), 10MWT increased by 84±66 m (p<0.001) and peak oxygen uptake (VO_2_) increased by 1.4±2.0 mL/kg/min (p=0.013). There were improvements in FSS score (−1.4±1.0, p<0.0001) and in most of the PROMIS-57 domains. The decrease in FSS scores correlated with an increase in the OCR/ECAR ratio (Pearson’s correlation r=-0.59, p=0.03). A subset of subjects (9/15) had significant reduction in their Interferon Stimulated Genes (ISG) (p=0.007) accompanied by a significant increase in the OCR/ECAR ratio (p=0.013).

**Conclusions:**

Cardiorespiratory function was improved in concomitance with reductions in fatigue following a 12-week aerobic exercise programme. The reduction in fatigue scores correlated with improvements in mitochondrial function.

## Introduction

One of the most debilitating symptoms of systemic lupus erythematosus (SLE) is excessive fatigue (SLE-fatigue), which persists even during periods of negligible disease activity or remission, is associated with physical activity intolerance and poor cardiopulmonary fitness.^1^ Cardiorespiratory dysfunction associated with fatigue may result from underlying pathophysiological processes and physiological deconditioning, other possible contributing factors include left ventricular dysfunction, pulmonary hypertension, interstitial lung disease and muscle atrophy.^2^

Aberrant mitochondrial function is shown to be associated with SLE-fatigue which improved on reversal of mitochondrial oxidative stress by repletion of glutathione by administration of N-acetylcysteine.^3^ Mitochondrial dysfunction also led to dysregulation in the type I interferon (IFN) pathway thereby promoting immune dysregulation, suggesting a putative role in SLE-fatigue.^4 5^ These studies suggest a putative role of mitochondrial dysfunction in SLE-fatigue.

The purpose of this exploratory study was to examine changes in objective measures of cardiorespiratory function and patient reported outcomes following an exercise training programme in women with SLE, and explore its association with measures of mitochondrial function. The primary outcome was time to attain the anaerobic threshold (AT-Time) during the cardiopulmonary exercise test (CPET). Secondary outcome measures were peak exercise capacity during the CPET and 10-minute walk test (10MWT) .

## Methods

### Study design

The study was approved by the National Institutes of Health (NIH) Institutional Review Board (ClinicalTrials.gov NCT03186794). Eligible subjects performed CPET and 10MWT, completed the Fatigue Severity Scale (FSS) and Patient Reported Outcomes Measurement Information System (PROMIS-57) survey, participated in exercise training and repeated all the assessments at the end.

### Subjects

Eligible subjects met the American College of Rheumatology (ACR) Revised Criteria for the Classification of SLE and had a Safety of Estrogen in Lupus Erythematosus, National Assessment modification of Systemic Lupus Erythematosus Disease Activity Index (SELENA-SLEDAI) score of ≤4 and FSS score ≥3. Sixteen subjects completed the study (online supplemental figure 1).

10.1136/lupus-2022-000778.supp1 Supplementary data

### Study procedures

#### Cardiopulmonary exercise test (CPET)

All subjects walked on a treadmill to an individualised increase in work rate projected to reach 8 METS in 10 min, with cardiorespiratory monitoring throughout the CPET (CardiO2 Ultima; MedGraphics Corp, St. Paul, Minnesota, USA). The target endpoint was volitional exhaustion by the subject. Anaerobic threshold (AT) was verified using accepted gas exchange analysis methods and peak oxygen consumption (peakVO_2_) was an average of the final 20 s of the CPET.^2^ Respiratory exchange ratio (RER) was calculated as the ratio of CO_2_ production to O_2_ consumption (VCO_2_/VO_2_).

#### Exercise training sessions

Supervised aerobic exercise training sessions comprised of treadmill walking, three times a week, over a period of 12 weeks, attending at least 29 out of 36 sessions. The goal was to complete 30 continuous minutes of vigorous intensity exercise (70%–80% of heart rate reserve).

### 10-Minute walk test (10MWT) and fatigability indices

A 10MWT was used to determine overground walking capacity by measuring the total distance walked in 10 minutes. Perceived and performance fatigability were measured using the methods of Schnelle and associates.^6^

#### Patient reported outcomes

The FSS evaluates the impact of fatigue on patients’ physical and social functioning and the PROMIS-57 survey captures quality of life over eight domains.

### Statistical analysis

Sample size was chosen based on feasibility and convenience, no formal determination were performed. Paired t-tests or Wilcoxon signed-rank test was used to determine if significant changes (post-training – pre-training) in the primary and secondary outcome measures were observed after the exercise training regimen. A Pearson’s correlation was calculated to examine the association between changes in FSS and OCR/ECAR ratio, baseline performance fatigability and change in performance fatigability at the end of study. A two-sided p value less than 0.05 was used to indicate statistically significant difference or relationship. Linear mixed effects models were used to analyse the longitudinal cytokines data, with visit as the fixed effect. An unstructured variance covariance matrix was used to account for the correlations among the repeated measures with the subject. For the comparisons of the cytokines between the pre-exercise visit and the post-exercise visit, the final p values were adjusted by the FDR (false discovery rate) method. Statistical analyses were performed in SAS (V.9.4, SAS Institute).

See online supplemental material for additional methods.

## Results

### Patient characteristics

Sixteen subjects completed the study and were included in the analyses. Almost all the subjects belonged to racial/ethnic minority groups (table 1). Disease activity and damage accrual at enrolment were low and no flares or changes in damage were observed during the study (Appendix—online supplemental table 1). The average prednisone dose was 5 mg and there were no changes made in medication regimens throughout the study.

**Table 1:** Baseline characteristics of subjects (n=16) completing the study

Age, years	42.0±10.3
Disease duration, years	9.1±6.5
BMI, kg/m^2^	28.2±5.2
Race/ethnicity, n (%)	
White	2 (12.5%)
African-American	3 (18.8%)
Asian	3 (18.8%)
Hispanic	8 (50%)
Gender, n (%)	
Female	16 (100%)
SELENA-SLEDAI	1.4±1.9
SLICC/ACR DI	0.8±1.2
Drugs, n (%)	
Glucocorticoids	11 (68.7%)
Antimalarials	15 (93.7%)
Azathioprine	5 (31.2%)
Methotrexate	2 (12.5%)
Mycophenolate mofetil	6 (37.5%)

Values are means±SD or count and percentage of total sample, n=number of patients.

BMI, body mass index; SELENA-SLEDAI, Safety of Estrogen in Lupus Erythematosus, National Assessment modification of Systemic Lupus Erythematosus Disease Activity Index; SLICC/ACR DI, Systemic Lupus International Collaborating Clinics/American College of Rheumatology Damage Index.

### Exercise capacity and fatigability

Subjects attended 92% of exercise training sessions (Appendix—online supplemental table 2) and spent 95% of their exercising time during these sessions at or above the target HR range There were no serious adverse events associated with exercise training.

**Figure 1:**
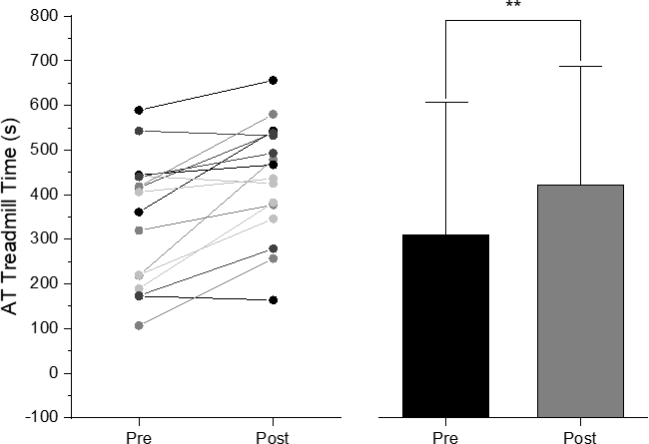
Time to reach the anaerobic threshold (AT-Time) at pre-training and post-training. Data are shown for each subject (symbols and lines) (n=16), with pre-training (black) and post-training (grey) comparisons shown as group means±SD. After 12 weeks of aerobic exercise training, subjects took longer to achieve the AT demonstrating improved fatigability (p<0.001).

At baseline CPET, 15 out of 16 subjects attained a HR ≥90% of age-predicted maximum HR (220 bpm—age) or peak RER≥1.10 (15/16 subjects) (table 2 and Appendix—online supplemental figure 2). Significant improvement in the primary outcome of AT-Time (+93 s, p<0.001) was observed after exercise training, as well as other outcomes at peak exercise (table 2 and figure 1) indicating overall improved cardiorespiratory function.

**Table 2:** Results of the CPET, patient reported outcomes and 10MWT (n=16)

	Pre-training	Post-training	Δ (post-pre)	P value
CPET variables (mean±SD)				
Peak exercise measures				
Treadmill time (s)	837 (203)	916 (153)	+79 (86)	0.002*
VO_2_ (mL/kg/min)	21.7 (3.5)	23.1 (3.8)	+1.4 (2.0)	0.013*
WR (W)	174 (55)	195 (46)	+21 (24)	0.004*
HR (beats/min)	157 (17)	154 (16)	−2 (12)	0.443
RER	1.18 (0.11)	1.17 (0.11)	−0.01 (0.07)	0.445
Submaximal exercise measures (at the anaerobic threshold)				
Treadmill time (s)	341 (144)	435 (130)	+93 (82)	< 0.001**
VO_2_ (mL/kg/min)	12.1 (2.4)	13.3 (2.6)	+1.2 (1.1)	<0.001**
HR (beats/min)	108 (15)	106 (10)	−2 (8)	0.193
WR (W)	44 (35)	68 (30)	+24 (21)	< 0.001**
Patient reported outcomes (mean±SD)				
FSS average all domains combined	4.6±1.2	3.1±1.4	−1.4±1.0	<0.0001**
10MWT				
Distance (m)	925 (131)	1009 (121)	+84 (66)	<0.001**
Performance fatigability index	1.11 (0.17)	0.99 (0.12)	−0.12 (0.09)	<0.001**
Perceived fatigability index	4.88 (1.92)	4.45 (2.11)	−0.43 (1.55)	0.501

*p=<0.05; **p=<0.001. Paired two-tailed, t-test were used for all results, except peak HR, VO_2_ for the AT, performance fatigability index and perceived fatigability index where a Wilcoxon signed-rank test was used. Shapiro-Wilk normality test was performed to evaluate normality assumption. No adjustments were made for multiple comparisons.

CPET, cardiopulmonary exercise test; FSS, Fatigue Severity Scale; HR, heart rate; 10MWT, 10-minute walk test; RER, respiratory exchange ratio; VO_2_, oxygen consumption; WR, work rate.

The average post-exercise training distance covered by subjects during 10MWT increased by 84 m (p<0.001) (table 2). A significant improvement in performance fatigability index (−0.12, p<0.001) was noted post-exercise training (table 2). An inverse relationship between the baseline and change in performance fatigability scores was noted (r=−0.747, p=0.001), suggesting that those experiencing the highest degrees of fatigability improved the most (online supplemental figure 3). The change in perceived fatigability index was not significant (−0.43, p=0.50).

### Patient reported outcomes

The composite FSS score was reduced after exercise training (−1.3, p<0.0001) (table 2). In addition, each of the individual FSS domains also significantly improved after exercise training (online supplemental table 3). There were significant improvements in most PROMIS-57 domains (Appendix—online supplemental table 4).^3 7^

### Mitochondrial responses

An inverse relationship between the post-training change in OCR/ECAR and FSS scores (r=−0.59, p=0.03) was observed (online supplemental figure 4).

## Discussion

The current study demonstrated significant improvements in objective measures of cardiorespiratory function and patient-reported fatigue following a vigorous treadmill walking programme in patients with SLE. Exploratory data suggested an improvement in mitochondrial function with an inverse correlation between fatigue and mitochondrial function following exercise training. The exercise programme was well tolerated with no serious adverse effects.

Peak VO_2_ (measured during a CPET) is accepted as gold standard measurements for cardiorespiratory function, while the anaerobic threshold (AT) is a marker of cardiorespiratory endurance. In the current study, there was an increase in peak VO_2_ of 7% which was lower compared with previous studies, perhaps as subjects in this study were older with higher damage accrual.^8^ The current study resulted in an increase in AT-Time and CPET duration after exercise training, indicating an improvement in metabolic function and exercise tolerance. Subjects also had large gains in the 10MWT distance and performance fatigability (table 2), these improvements were greatest in subjects with the worst performance fatigability at baseline (online supplemental figure 3).

Consistent with previous studies, participants reported a significant improvement in fatigue and physical function.^8–10^ Select domains of PROMIS-57 did not change in this study (ie, anxiety, satisfaction with social roles and pain). Previous studies have also shown improvement in physical fatigue, physical role and vitality domains of 36-Item Short Form Survey (SF-36), but unlike our study, there was no improvement in domains of depression and sleep. Indeed, conflicting results for psychosocial and cognitive function outcomes are reported in the literature, suggesting additional regulators of SLE-fatigue such as psychological factors and pain, may need to be addressed.^8 9 11^

Mitochondrial dysfunction has been reported in SLE and proposed as a putative biological mechanism for chronic fatigue.^4^ In the current study, we observed an inverse correlation between mitochondrial function and SLE-fatigue with exercise training, and a post-exercise improvement in mitochondrial dysfunction. These data suggest mitochondrial dysfunction as perhaps one of the determinants and a therapeutic target for SLE-fatigue.^10^

Our study has limitations. We did not include control group of subjects with or without SLE. However, our main outcome variable was objective measure of adaptation to aerobic exercise training and less likely to be subjected to observational bias. Our study included a small number of subjects, all of whom were highly motivated, which is in contrast to a survey showing only 28% of patients with SLE engage in moderate-to-vigorous intensity physical activity.^12^

In summary, this single treatment-arm study of supervised exercise training demonstrated improvements in objective measures of exercise capacity and patient reported fatigue. Novel insights into mitochondrial dysfunction and their potential improvement after exercise training were also underscored. The study has emphasised associations among improvements in cardiorespiratory function, performance fatigability and SLE-fatigue, and provides further evidence that exercise training may be an effective adjunct to treatment in women with SLE.
