# Supplementary material for: Changes in cardiorespiratory function and fatigue following 12 weeks of exercise training in women with systemic lupus erythematosus: a pilot study
Source: Lupus Sci Med. 2022 Oct 11;9(1):e000778. doi: 10.1136/lupus-2022-000778 (PMC9557301; doi:10.1136/lupus-2022-000778)
Supplement: Supplementary data [file lupus-2022-000778supp001.pdf]

Supplemental Material

Supplemental Methods:

Inclusion Criteria

- Fulfilling 4 of the 11 American College of Rheumatology Criteria for the Classification of Systemic Lupus Erythematosus
- Age 21 to 80
- Female Gender
- BMI less than 40
- No primary or secondary medical conditions that would limit aerobic capacity or make exercise participation unsafe. These conditions are found under the exclusion criteria listed below and include cardiovascular disease and cardiomyopathy, pulmonary and pulmonary vascular disease, stroke, significant hepatic or renal dysfunction, most cancers, diabetes mellitus, HIV infection, and peripheral vascular disease.
- SELENA-SLEDAI score ≤ 4, maintained for at least three months. (C3 and C4 levels are measured as markers for stability and included in the SELENA-SLEDAI score if abnormal).
- No increase in doses of immunosuppressive medications (hydroxychloroquine, mycophenolate mofetil, azathioprine, methotrexate) for at least three months at the time of screening.
- No increase in the dose of prednisone or equivalent steroid in the past 3 months at the time of screening.
- Physically inactive, not participating in aerobic exercise training at heart rate above 60% maximum heart rate, 20 min/session or more, 2 or more days per week, within the last 6 months at the time of screening.
- FSS composite score ≥ 3 indicating the presence of clinically significant fatigue
- Subjects must be able to walk on a treadmill.

Exclusion Criteria

- Prednisone ≥ 15 mg daily (or equivalent)
- Have started azathioprine, mycophenolate mofetil, methotrexate, cyclophosphamide or biologics within 3 months
- Rituximab infusion within 6 months
- Present symptoms of ischemic heart disease, right- or left-sided heart failure, cor pulmonale or pulmonary hypertension, dilated or hypertrophic cardiomyopathy or non- idiopathic cardiomyopathy
- Significant pulmonary dysfunction (obstructive, restrictive, or infectious pulmonary disease)
- Significant hepatic(LFT > 2 times of upper limit of normal) or renal dysfunction (GFR<45 ml/min)
- Deep vein thrombosis
- Chronic anticoagulation, (with the exception of low dose aspirin), or a history of a bleeding disorder
- History or presence of any form of cancer other than skin cancer or cervical in-situ cancer
- History of cerebrovascular accident
- Orthopedic conditions that would limit performance of treadmill exercise tests or treadmill exercise training

- Current smoker or active substance abuse
- HIV infection

Ten-minute walk test (10MWT) and Fatigability Indices:

A 10MWT was used to determine overground walking capacity and to provide a mechanism from which performance and perceived fatigability scores could be calculated. The test was conducted by having subjects walk around an 80-meter circular course and subjects were instructed to walk as far as they could in ten minutes, during which velocity measurements were obtained at 2.5-minute intervals. The 10MWT was scored by measuring the total distance walked in 10-minutes. Perceived and performance fatigability were measured using the methods of Schnelle and associates (1). The performance fatigability index was calculated by dividing the average velocity of the entire 10-minutes of the test into the average velocity achieved over the first 2.5 minutes (decline in performance), then further dividing the previous performance quotient by the total distance walked (an index of intensity) (1):

$$[(\bar{v}_{10mins}/\ \bar{v}_{2.5mins})\ /10MWT\ distance]\ x\ 1000$$

Perceived fatigability index was simultaneously evaluated by having the subject rate their perceived changes in the feeling of tiredness over the 10-minutes of the test on a numerical visual response scale and dividing the change by the total distance walked (1):

(10 MWT post fatigability rating/ 10 MWT distance) x 1000.

Health Related Quality of Life and Fatigue Measures:

The PROMIS–57 Profile v2.0 and FSS were collected at baseline and post-exercise training. The FSS is a subjective method of evaluating the impact of fatigue on patients' physical and social functioning and has been validated in patients who have SLE (2). The FSS is a short 9-item questionnaire that requires patients to rate the severity of their fatigue (on a 7-point Likert scale) over the past week. The FSS composite score was calculated as an average of all 9-items and higher scores were indicative of greater fatigue.

The PROMIS-57 Profile (v2.0) captures quality of life and consists of a collection of short forms covering 8 domains (a total of 57 questions) assessing anxiety, depression, fatigue, pain interference, physical function, sleep disturbance, and ability to participate in social roles and activities, and pain intensity item (3, 4). All domains except pain are scored on a 5-point Likert Scale, whereas pain is scored on a numerical rating scale ranging from 0 to 10. Scores for all domains were calculated by converting the raw scores into a standardized T-score with a mean of 50 and a standard deviation (SD) of 10, except the pain intensity domain which was reported as a raw score. Domains were not weighted and higher PROMIS scores reflect more of the domain being measured; thus, higher scores in domains measuring symptoms reflect worse symptoms but higher scores in domains measuring function reflect better functioning.

Mitochondrial function extracellular flux measurement:

The parameters assessed using extracellular flux were measured as previously described (5, 6). The peripheral blood mononuclear cells (PBMCs) were isolated from blood samples within 2 hours of collection, and plated at $1.5x10^5$ cells per well to reach 80-90% confluency(5).

Mitochondrial function parameters assessed using extracellular flux were measured using peripheral blood mononuclear cells (PBMCs) that were isolated from human peripheral blood samples within 2 hours of collection using the BD Vacutainer CPT Mononuclear Cell Preparation Tubes based on manufacturer's protocol (BD Biosciences; Becton, Dickinson and Company). Cell plates were coated before each experiment with freshly reconstituted Cell-Tak cell and tissue adhesive solution (Corning, Inc.). PBMCs were plated at 1.5x105 cells per well to reach 80-90% confluency. Respiratory inhibitors (Agilent Technologies, Inc.) were reconstituted within 1

hour prior to the assay: Oligomycin - 1 μM, carbonyl cyanide-4-(trifluoromethoxy)phenylhydrazone - 1 μM and antimycin A/rotenone - 0.5 μM. Cells were plated in Cell-Tak-coated plates and equilibrated in a non-CO2 incubator for 45-60 min in the assay media.Cell plates were then inserted into a Seahorse XFp extracellular flux instrument (Agilent Technologies, Inc.). Respiratory inhibitors were injected sequentially (Oligomycin, FCCP, antimycin A/rotenone) into the corresponding ports and the oxygen consumption rate as well as extracellular acidification rate were measured at baseline and following each drug injection. To normalize the functional data, live cells were stained with the Cell Proliferation Assay (Thermo Fisher Scientific, Inc.) and quantified using the Cytation 1 instrument (Biotek Instruments, Inc.). Oxygen consumption rate metabolic potential was calculated as: O2 (pMol/min) [FCCP] ÷ O2 (pMol/min) [baseline]. Extracellular acidification rate metabolic potential was calculated as: Proton concentration (mpH/min) [FCCP] ÷ Proton concentration (mpH/min) [baseline].

Serum Cytokine Measurement:

Serum was collected from all subjects and measured using ProcartaPlex multiplex immunoassays (Thermo Fisher Scientific) according to the manufacturer's instructions. The concentrations of the cytokines were calculated according to the standard curve of the assay. The concentrations of the cytokines were statistically analyzed using the Mann-Whitney test, and plotted with GraphPad Prism software package(7).

RNA Isolation and Quantification of type I Interferon Stimulated Gene Signature (ISGs) by Nanostring:

Total RNA was extracted from whole blood using Paxgene blood RNA isolation kit (PreAnalytiX. Switzerland). A target-specific oligonucleotide probe pairs (synthesized by IDT, Coralville, IA) contained 37 IFN Regulatory Genes discriminative of the IFN signature and 4 housekeeping genes (ALAS1, HPRT1, TBP, TUBB) detailed methods as previously described(7).

Supplementary Results:

Mitochondrial responses and ISGs: There were a significant improvement in the ISG in 60% (9/15) of the subjects at the end of exercise training, (calculated from 37 ISGs scores; p=0·009) (Supplemental Figure 5) (8, 9). The subjects with improved post-exercise ISG also exhibited a statistically increase in OCR/ECAR ratio following exercise (p=0·013), however the increase was mostly driven by one outlier subject (Supplemental Figure 6).

All 9 subjects with improved ISG had an increase in the cardiorespiratory function, improved fatigue and adhered to the exercise training. However only 6 of these subjects had complete OCR/ECAR across timepoints to allow for assessment of changes in OCR/ECAR ratios from pre- to post-exercise.

Serum Cytokines: There were significant changes in plasma concentrations of serum cytokines, IL-7, IL-22 and MMP, but after adjusting for multiple comparisons none of the FDR adjusted p-values remained significant (Supplemental Table 5).

Supplemental Table 1: Results of the SELENA-SLEDAI and SLICC/ACR DI Indices (N=16)

**Disease Activity and Damage (Mean ±SD)**	**Pre-exercise**	**Post-exercise**	**Pre-Post change**	***p-Value***
**SLICC/ACR DI**	0.8 ± 1.2	0.9 ± 1.3	0.1 ± 0.3	0.33
**SELENA-SLEDAI**	1.4 ± 1.9	1.2 ± 1.6	-0.2 ± 1	1

Abbreviations:

SELENA-SLEDAI: Safety of Estrogen in Lupus Erythematosus, National Assessment modification of Systemic Lupus Erythematosus Disease Activity Index. SLICC/ACR DI: Systemic Lupus International Collaborating Clinics / American College of Rheumatology damage index Paired two-tailed, t-test were used for all results. Shapiro-Wilk Normality Test of group differences were performed to evaluate normality assumption. No adjustments were made for multiple comparisons.

Supplemental Table 2. Summary of the Aerobic Exercise Training Intervention

**# of Sessions**	**Training HR Range**		**Average Training HR**	**% HRR**	**Average RPE**
	**70% HRR**	**80%HRR**			
33 (3)	133 (14)	141 (15)	136 (13)	74 (3)	4.2 (0.9)

Values are mean (±SD). Abbreviations: HR, heart rate; HRR, heart rate reserve; RPE, Ratings of Perceived Exertion using Borg's Category Ratio Scale (0 to 10 scale).

Supplemental Table 3. Results of the Individual Domains of Fatigue Severity Scale (FSS) (N=16)

**FSS Domains (Mean ± SD)**	**Pre-exercise**	**Post-exercise**	**Δ (Post-pre)**	***p-value***
My motivation is lower when I am fatigued	5·5 ± 1·4	4·4 ± 2·2	-1·1 ± 1·8	0·02*
Exercise brings on my fatigue	4·6 ± 1·3	2·4 ± 1·4	-2·1 ± 1·6	0·0001**
I am easily fatigued	4·4 ± 1·2	3·4 ± 1·6	-0·9 ± 1·4	0·02*
Fatigue interferes with my physical functioning	4·5 ± 1·5	3·1 ± 1.5	-1·4 ± 1·3	0·0005**
Fatigue causes frequent problems for me	4·4 ± 1·8	2·6 ± 1·6	-1·8 ± 1·6	0·0004**

My fatigue prevents sustained physical functioning	3·9 ± 1·9	2·7 ± 1·6	-1·3 ± 1·8	0·01*
Fatigue interferes with carrying out certain duties and responsibilities	4·4 ± 2·2	2·8 ± 1·8	-1·6 ± 1·9	0·003*
Fatigue is among my three most disabling symptoms	5 ± 1·9	3·3 ± 2	-1·7 ± 2·1	0·006*
Fatigue interferes with my work, family, or social life	4·3 ± 2·1	3·4 ± 1·9	-0·9 ± 1·4	0·02*
FSS Average All Domains Combined	4·6 ± 1·2	3·1 ± 1·4	-1·4 ± 1·0	<0·0001**

Abbreviation: FSS: Fatigue Severity Scale The numbers are mean ± SD of as reported by subjects based on their symptoms during the past week on a continuous scale from 1-7. A score of 1 indicate they disagree and score of 7 indicates that they agree. * p<0·05 **p<0·001. Paired two-tailed, t-test were used for all results. Shapiro-Wilk Normality Tests were performed to evaluate normality assumption.

Supplemental Table 4: Results of the Individual PROMIS-57 Domains (N=16)

PROMIS-57 Domains (Mean ± SD)	Pre-exercise	Post-exercise	Pre-Post change	*p-value*
**Physical Function**	46.8 ± 5.0	50.4 ± 6.9	3.6 ± 4.8	0.01
**Anxiety**	52.0 ± 9.8	48.5 ± 9.8	-3.5 ± 7.0	0.06
**Depression**	49.9 ± 5.9	45.2 ± 8.0	-4.8 ± 6.4	0.01
**Fatigue**	52.4 ± 5.8	46.5 ± 6.9	-6.0 ± 4.5	<.0001
**Sleep Disturbance**	52.1 ± 6.1	49.7 ± 6.1	-2.4 ± 4.3	0.04
**Ability to Participate in Social Roles and Activities**	54.3 ± 7.5	55.3 ± 7.5	1.0 ± 5.3	0.45
**Pain Interference**	51.3 ± 9.5	47.4 ± 7.5	-3.9 ± 10.8	0.17
**Pain Intensity**	2.6 ± 2.0	1.7 ± 1.3	-0.9 ± 1.8	0.06

Abbreviation: PROMIS-57: Patient Reported Outcomes Measurement Information System with total of 57 questions in 8 domains.

All domains except pain are scored on a 5-point Likert Scale, whereas pain is scored on a numerical rating scale ranging from 0-10. The scores from 7 domains using Likert scale were calculated with a conversion of the raw score into a standardized T-score with a mean of 50 and a standard deviation (SD) of 10, the pain intensity domain was reported as raw score. The higher PROMIS scores reflect more of the domain being measured; thus, higher scores in

domains measuring symptoms reflect worse symptoms but higher scores in domains measuring function reflect better functioning. *= p=<0.05 **p=<0.001

Paired two-tailed, t-test were used for all results. Shapiro-Wilk Normality Test of group differences were performed to evaluate normality assumption.

Supplemental Table 5: Serum Cytokines

**Cytokines (pg/mL)** **Mean (SD)**	**Pre-Exercise Training**	**Mid-point of exercise training**	**Post-Exercise training**	**Raw p-value**	**FDR adjusted p-value**
APRIL	1694.98 (4319.69)	1579.09 (3940.22)	1667.09 (4314.27)	0.71	0.86
CCL3	29.8 (30.31)	28.99 (29.37)	29.08 (27.82)	0.94	0.95
CCL4	18.8 (17.71)	17.92 (15.79)	18.2 (15.69)	0.61	0.82
CCL7	10.16 (14.15)	10.41 (13.75)	10.94 (18.53)	0.32	0.77
CCL8	8.92 (5.44)	8.64 (5.28)	8.24 (4.31)	0.51	0.77
CCL20	28.94 (32.08)	29.75 (33.08)	35.7 (56.15)	0.63	0.82
CCL24	137.27 (55.7)	129.35 (78.67)	129.87 (56)	0.25	0.77
CD30	283.86 (191.46)	270.37 (193.78)	299.91 (204.12)	0.58	0.82
CD40L	119.25 (114.53)	132.96 (158.21)	118.68 (112.17)	0.85	0.95
CSF3	6.54 (11.98)	6.88 (12.03)	7.44 (10.91)	0.34	0.77
CX3CL1	16.91 (58.45)	14.81 (49.77)	15.49 (53.21)	0.28	0.77
CXCL1	2.14 (4.86)	3 (6.26)	2.84 (5.68)	0.46	0.77
CXCL5	243.58 (266.35)	259.37 (353.88)	229.3 (197.12)	0.87	0.95
CXCL11	62.92 (206.57)	65.83 (192.99)	74.32 (230.04)	0.16	0.77
CXCL13	314.74 (216.58)	330.22 (224.23)	281.63 (242.38)	0.46	0.77
Eotaxin	32.35 (25.74)	29.3 (24.7)	32.98 (30.55)	0.52	0.77
GMCSF	17.99 (47.94)	15.68 (38.66)	19.42 (53.24)	0.23	0.77
HGF	57.43 (36.83)	58.39 (42.49)	50.89 (31.28)	0.37	0.77
IFNa	2.74 (3.49)	2.84 (3.57)	3.49 (5.49)	0.07	0.77
IFNg	8.64 (13.31)	9.12 (15.5)	9.67 (17.38)	0.62	0.82
IL2	60.54 (166.63)	54.3 (142.93)	64.86 (185.67)	0.45	0.77
IL3	3481.73 (14308.49)	2027.78 (8314.51)	2518.85 (10327.44)	0.20	0.77
IL4	22.95 (43.16)	24.24 (48.87)	29.73 (66.05)	0.80	0.95
IL5	23.91 (38.75)	23.26 (37.02)	25.35 (42.1)	0.60	0.82
IL6	26.45 (47.37)	26.49 (43.98)	27.86 (56.28)	0.51	0.77

IL7	1.17 (1.04)	0.99 (0.81)	0.91 (0.72)	0.01	0.18
IL8	3.52 (3.5)	3.27 (3.06)	4.79 (7.59)	0.52	0.77
IL9	4.51 (2.58)	4.7 (3.13)	4.44 (3.01)	0.44	0.77
IL13	1.65 (1.77)	1.61 (1.64)	1.94 (2.25)	0.19	0.77
IL15	15.74 (19.84)	15.35 (19.5)	16.29 (22.25)	0.62	0.82
IL16	42.63 (15.41)	44.03 (19.61)	41.49 (18.63)	0.40	0.77
IL18	20.15 (18.9)	18.21 (19.2)	18.02 (16.07)	0.26	0.77
IL20	25.36 (35.77)	26.73 (38.16)	24.57 (32.97)	0.51	0.77
IL21	73.8 (266.12)	55.08 (191.4)	82.02 (291.48)	0.37	0.77
IL22	258.27 (956.7)	205.02 (738.78)	322.73 (1184.16)	0.04	0.76
IL27	91.51 (222.71)	89.77 (230.51)	114.7 (316.55)	0.70	0.86
IL12p70	2.7 (1.49)	2.76 (1.66)	3.12 (2.76)	0.12	0.77
IL17A	235.47 (240.15)	211.69 (226.89)	212.94 (229.8)	0.85	0.95
IL1a	1.15 (2.51)	1.1 (1.83)	1.07 (2.21)	0.42	0.77
IL1b	8.95 (14.51)	9.01 (14.2)	10.08 (16.82)	0.14	0.77
IL2R	813.55 (650.92)	787.24 (664.27)	892.42 (751.87)	0.65	0.82
IP10	30.23 (15.58)	30.75 (17.37)	29.75 (15.03)	0.84	0.95
LIF	11.39 (10.36)	10.94 (9.55)	11.3 (10.45)	0.32	0.77
MCP1	90.57 (67.25)	88.15 (76.51)	102.46 (75.19)	0.41	0.77
MDC	102.6 (69.9)	105.37 (69.5)	113.67 (74.85)	0.14	0.77
MIF	26.58 (11.62)	32.4 (26.18)	28.47 (12.98)	0.31	0.77
MIG	287.57 (1137.38)	235.1 (927.19)	269.58 (1065.18)	0.35	0.77
MMP1	230.69 (255.77)	262.06 (361.5)	283.14 (341.46)	0.00	0.18
NGFb	1.71 (2.02)	1.71 (2.19)	2.29 (4.37)	0.92	0.95
SCF	7.39 (6.09)	6.95 (5.72)	7.45 (6.61)	0.98	0.98
SDF1a	1161.34 (1932.89)	1117.04 (1950.39)	1133.85 (1940.31)	0.29	0.77
TNFRII	108.42 (149.39)	100.77 (134.16)	104.43 (143.92)	0.09	0.77
TNFa	2.69 (4.58)	2.66 (4.16)	3.92 (9.48)	0.33	0.77
TNFb	14.77 (22.9)	14.51 (21.09)	18.25 (35.96)	0.34	0.77
TRAIL	465.57 (1634.98)	407.26 (1415.13)	433.42 (1528.89)	0.92	0.95
TSLP	21.82 (32.77)	22.77 (36.81)	24.1 (39.51)	0.94	0.95
TWEAK	1561.35 (1124.22)	1587.37 (1317.44)	1535.88 (973.49)	0.67	0.84
VEGFa	490.94 (924.18)	422.97 (778.29)	486.96 (913.02)	0.89	0.95
BAFF	52.53 (90.9)	57.55 (111.37)	63.06 (130.87)	0.42	0.77
CCL26	0.37 (0)	0.37 (0)	0.91 (1.92)	0.50	0.77

FGF2	1.49 (1.27)	1.52 (1.38)	3.66 (10.2)	0.31	0.77
IL10	5.04 (9.01)	4.45 (6.47)	5.75 (10.04)	0.30	0.77
IL23	18.73 (8.8)	18.23 (6.74)	22.48 (24.29)	0.31	0.77
IL31	15.87 (15.46)	15.83 (15.27)	19.63 (30.95)	0.31	0.77
MCSF	81.7 (52.95)	78.11 (37.54)	124.27 (228.46)	0.31	0.77

Changes in serum cytokines concentration comparing pre-exercise values with mid-exercise training values, and post-exercise values for any significant changes. The repeated measures data were analyzed by the linear mixed models, p-values for the linear contrasts were calculated and corrected using the FDR (false discovery rate) method.

Supplemental Figure 1

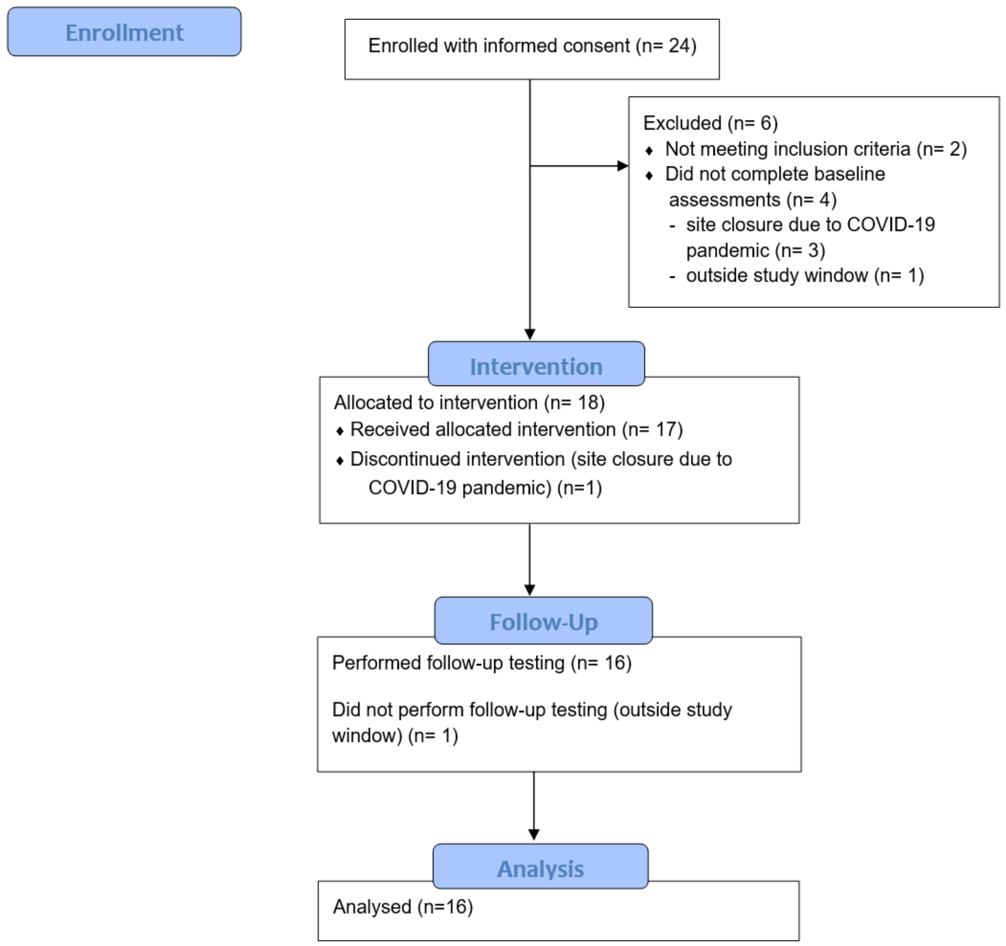

Supplemental Figure 1: CONSORT Diagram

Supplemental Figure 2

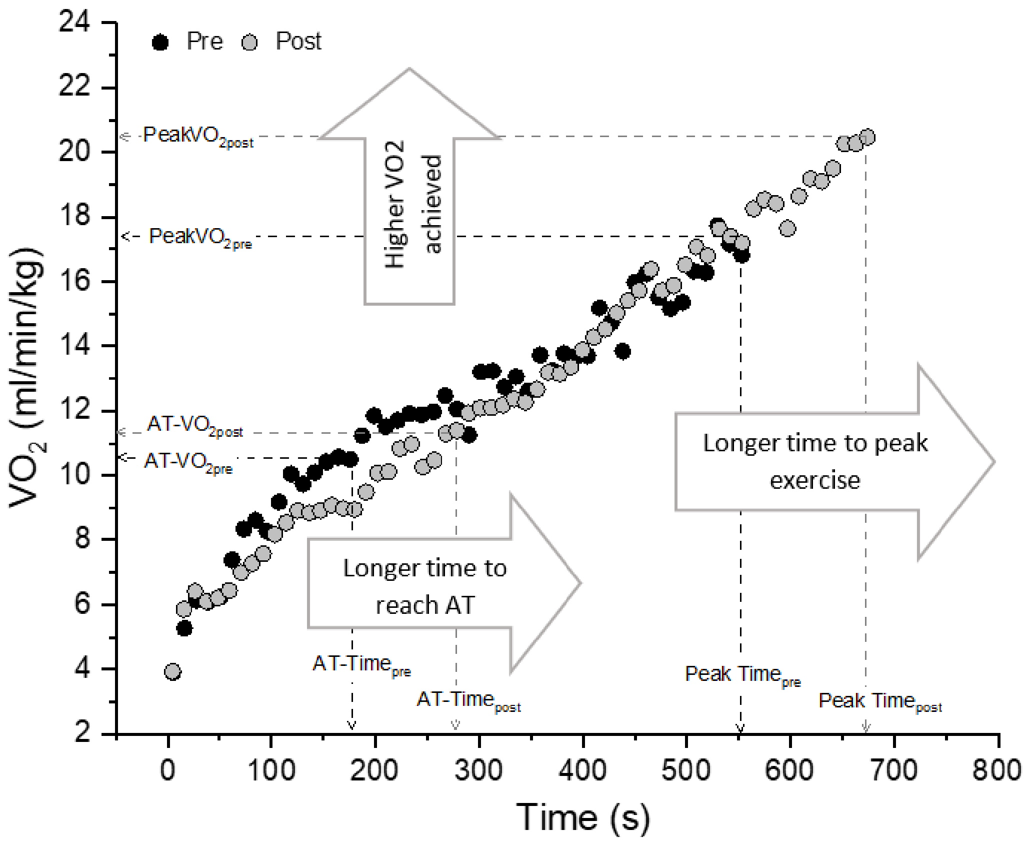

Supplemental Figure 2: Summary of the changes observed during a Cardiopulmonary Exercise Test (CPET) with exercise training. Data for a representative subject for pre-training (black) and post-training (grey) are shown. Treadmill grade and speed are gradually increased over time to achieve a near-linear increase in work rate, observed as a corresponding increase in VO2 throughout the CPET. The time and VO2 associated with the anaerobic threshold (AT) and peak for pre and post-training is shown as dashed lines.

Supplemental Figure 3

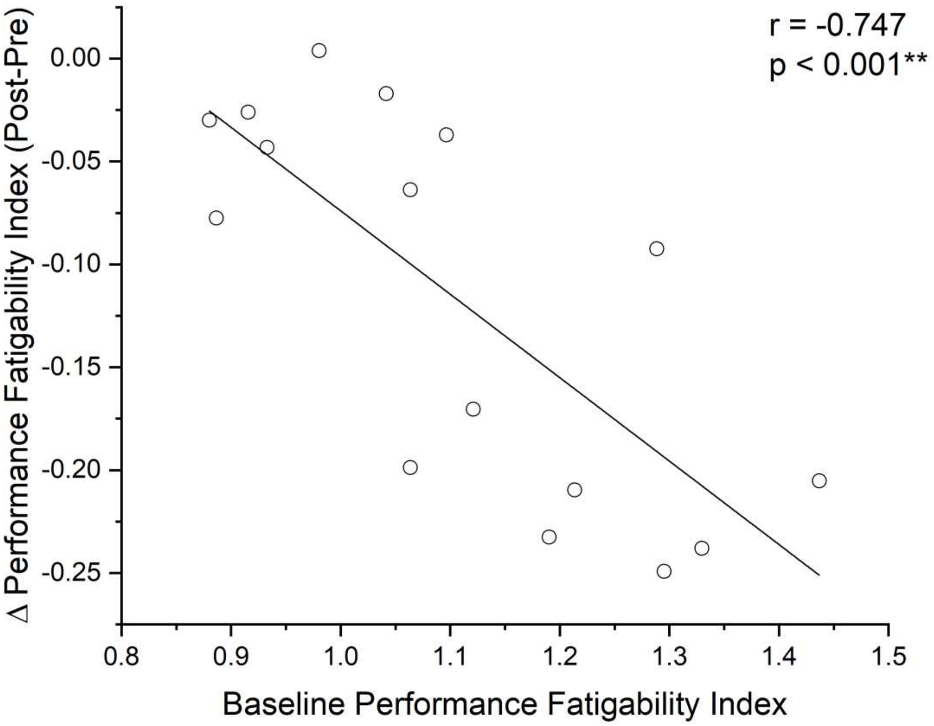

Supplemental Figure 3: Association between baseline performance fatigability and change in performance fatigability after 12 weeks of aerobic training:

Subjects (n=16) reporting greater fatigability at the start of the exercise program were observed to have the most improvement following 12 weeks of aerobic training (r=-0·747, p<0·001).

Supplemental Figure 4

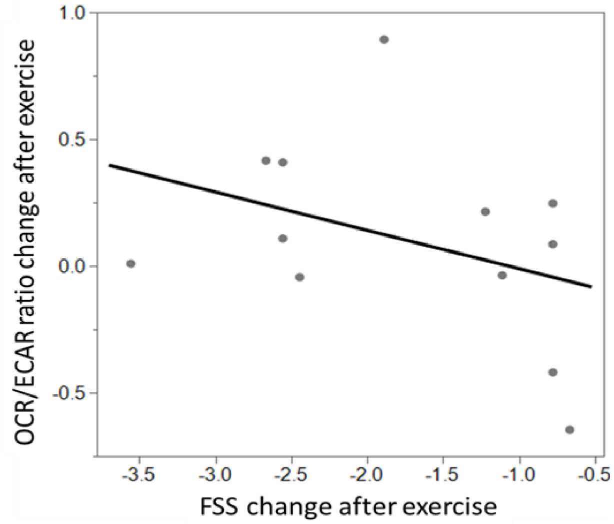

Supplemental Figure 4: Changes in Oxygen consumption Rate (OCR) / ExtraCellular Acidification Rate (ECAR) and Fatigue Severity Scale (FSS).

Post-exercise changes in Fatigue Severity Scale (FSS) compared with changes in Oxygen consumption Rate (OCR) / ExtraCellular Acidification Rate (ECAR) ratio (N=12) (samples from 4 subjects were unusable due to failed quality control). The difference from baseline to post-exercise in FSS was inversely correlated with the differences in OCR/ECAR ratios (r = -0·59, p = 0·03).

Supplemental Figure 5

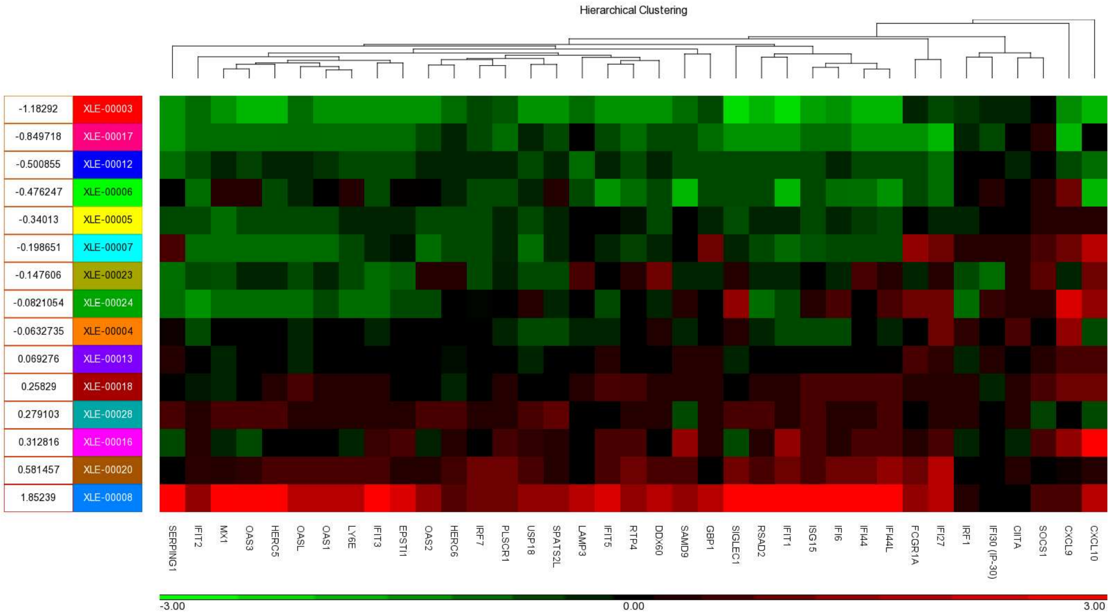

Supplemental Figure 5: Heatmap of 37 Interferon Stimulated Gene Signature (ISGs) (N=15, samples from one subject were lost). Expression of 37 Interferon Responsive Genes previously identified as discriminative of the Interferon Stimulated gene signature (ISGs) in peripheral blood by Nanostring. Heat map shows Log2 fold change for these genes between pre-exercise training vs post-exercise training for each subject/gene, sorted by ascending ratio of ISGs. Post-exercise training 9/15 subjects had a significant improvement in their ISGs.

Supplemental Figure 6

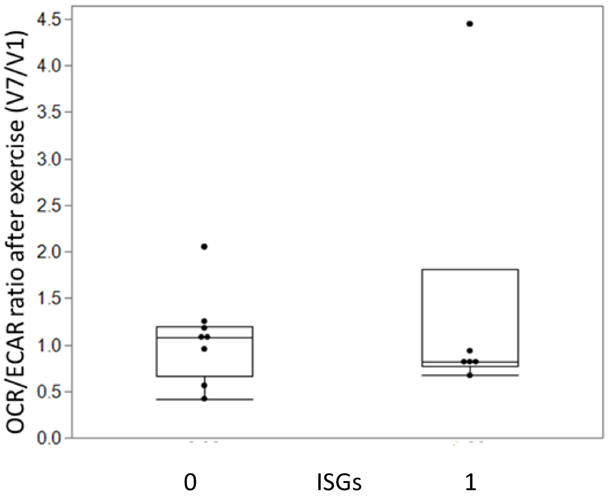

Supplemental Figure 6: Changes in Oxygen consumption Rate (OCR) / ExtraCellular Acidification Rate (ECAR) and Interferon Stimulated Gene Signature (ISGs).

Oxygen Consumption Rate (OCR) / ExtraCellular Acidification Rate (ECAR) ratio in subjects with improved Interferon Stimulated Gene Signature (ISGs) =1 (N=6) and subjects without improved ISGs=0 (N=8) (samples from 2 subjects were unusable due to failed quality control). Subjects with improved IFN signature (1) exhibited higher OCR/ECAR ratios post-exercise compared to baseline vs. patients without improved IFN signature (0) ($p = 0{\cdot}013$).

References:

1. Schnelle JF, Buchowski MS, Ikizler TA, Durkin DW, Beuscher L, Simmons SF. Evaluation of two fatigability severity measures in elderly adults. J Am Geriatr Soc. 2012;60(8):1527-33.

2. Krupp LB, LaRocca NG, Muir J, Steinberg AD. A study of fatigue in systemic lupus erythematosus. J Rheumatol. 1990;17(11):1450-2.
3. Kasturi S, Szymonifka J, Burket JC, Berman JR, Kirou KA, Levine AB, et al. Validity and Reliability of Patient Reported Outcomes Measurement Information System Computerized Adaptive Tests in Systemic Lupus Erythematosus. J Rheumatol. 2017;44(7):1024-31.
4. Cella D, Riley W, Stone A, Rothrock N, Reeve B, Yount S, et al. The Patient-Reported Outcomes Measurement Information System (PROMIS) developed and tested its first wave of adult self-reported health outcome item banks: 2005-2008. J Clin Epidemiol. 2010;63(11):1179-94.
5. Feng LR, Nguyen Q, Ross A, Saligan L. Evaluating the Role of Mitochondrial Function in Cancer-related Fatigue. Journal of visualized experiments : JoVE [Internet]. 2018; 135:[57736 p.].
6. Feng LR, Wolff BS, Liwang J, Regan JM, Alshawi S, Raheem S, et al. Cancer-related fatigue during combined treatment of androgen deprivation therapy and radiotherapy is associated with mitochondrial dysfunction Corrigendum in /10.3892/ijmm.2020.4546. Int J Mol Med. 2020;45(2):485-96.
7. Hasni SA, Gupta S, Davis M, Poncio E, Temesgen-Oyelakin Y, Carlucci PM, et al. Phase 1 double-blind randomized safety trial of the Janus kinase inhibitor tofacitinib in systemic lupus erythematosus. Nat Commun. 2021;12(1):3391.
8. Furie R, Khamashta M, Merrill JT, Werth VP, Kalunian K, Brohawn P, et al. Anifrolumab, an Anti-Interferon-α Receptor Monoclonal Antibody, in Moderate-to-Severe Systemic Lupus Erythematosus. Arthritis Rheumatol. 2017;69(2):376-86.
9. Yao Y, Higgs BW, Morehouse C, de Los Reyes M, Trigona W, Brohawn P, et al. Development of Potential Pharmacodynamic and Diagnostic Markers for Anti-IFN-α Monoclonal Antibody Trials in Systemic Lupus Erythematosus. Hum Genomics Proteomics. 2009;2009.
